# Prohibitin involvement in the generation of mitochondrial superoxide at complex I in human sperm

**DOI:** 10.1111/jcmm.12945

**Published:** 2016-08-25

**Authors:** Ran‐Ran Chai, Guo‐Wu Chen, Hui‐Juan Shi, Wai‐Sum O, Patricia A. Martin‐DeLeon, Hong Chen

**Affiliations:** ^1^ Department of Anatomy, Histology & EmbryologyKey Laboratory of Medical Imaging Computing and Computer Assisted Intervention of ShanghaiInstitute of Reproduction and DevelopmentShanghai Medical CollegeFudan UniversityShanghaiChina; ^2^ Shanghai Ji Ai Genetics and IVF InstituteObstetrics and Gynecology HospitalInstitute of Reproduction and DevelopmentFudan UniversityShanghaiChina; ^3^ Institute of Reproduction and DevelopmentShanghai Medical CollegeFudan UniversityNational Population and Family Planning Key Laboratory of Contraceptive Drugs & DevicesShanghai Institute of Planned Parenthood ResearchShanghaiChina; ^4^ School of Biomedical SciencesThe University of Hong KongHong Kong SARChina; ^5^ Department of Biological SciencesUniversity of DelawareNewarkDEUSA

**Keywords:** prohibitin, mitochondrial ROS, mitochondrial complex I, sperm motility, human sperm

## Abstract

Prohibitin (PHB), a major mitochondrial membrane protein, has been shown earlier in our laboratoryto regulate sperm motility *via* an alteration in mitochondrial membrane potential (MMP) in infertile men with poor sperm quality. To test if PHB expression is associated with sperm mitochondrial superoxide (mROS) levels, here we examined sperm mROS levels, high MMP and lipid peroxidation in infertile men with poor sperm motility (asthenospermia, A) and/or low sperm concentrations (oligoasthenospermia, OA). The diaphorase‐type activity of sperm mitochondrial complex I (MCI) and PHB expression were also determined. We demonstrate that mROS and lipid peroxidation levels are significantly higher in sperm from A and OA subjects than in normospermic subjects, whereas high MMP and PHB expression are significantly lower. A positive correlation between mROS and lipid peroxidation and a negative correlation of mROS with PHB expression, high MMP, and sperm motility were found in these subjects. The finding of similar diaphorase‐type activity levels of sperm MCI in the three groups studied suggests that the catalytic subunits of MCI in the matrix arm may produce mROS on its own. There may be a dysfunction of electron transport at MCI associated with decreased expression of PHB in sperm with poor quality. We conclude that mROS level is increased and associated with decreased PHB expression, and it may regulate sperm motility *via* increases in low MMP and lipid peroxidation. This is the first report on the involvement of PHB in human sperm motility loss associated with increased generation of mROS at MCI.

## Introduction

Reactive oxygen species (ROS), such as superoxide anions, hydroxyl radicals and H_2_O_2_, can be produced during cellular metabolism and are maintained at physiological levels by endogenous enzymatic and non‐enzymatic antioxidants. For example in somatic cells, the major source of ROS generation is electron leakage from mitochondrial electron transport chain (ETC) during cellular respiration 1. However, once the fine balance controlled by antioxidant factors within the inter‐membrane space and mitochondrial matrix 2 is lost, mitochondrial ROS production may generate oxidative stress that has been implicated in numerous pathological conditions, including Alzheimer's 3 and Parkinson's disease 4. Current evidence supports the generation of ROS by sperm mitochondria in rabbits 5, 6, rats 7 and human 8, but the factors responsible for excessive mitochondrial free radical generation remains unidentified.

Human mature sperm contain 22–28 mitochondria helically arranged around the mid‐piece axoneme, where they play a key role in the maintenance of sperm motility by the generation of optimal levels of ROS 8 and ATP 9, 10. Prohibitin (PHB) is a highly conserved major mitochondrial inner membrane protein and has been shown to be important for maintaining mitochondrial function in lower organisms by exerting a chaperone‐like function for handling mitochondrial membrane proteins and/or structural scaffolds and stabilizing the OXPHOS system 11. In addition, its deficiency in somatic cells is associated with mitochondrial membrane depolarization and increased generation of ROS 12. Our recent findings of a decrease in PHB expression in infertile men with poor sperm quality, accompanied by an alteration in mitochondrial membrane potential (MMP) 13, suggested that PHB may play an important role in mitochondrial function of human sperm. However, its relationship with mitochondrial ROS (superoxide) generation in human sperm mitochondria remains elusive.

To test if PHB expression is associated with sperm mitochondrial ROS (mROS) level, we have investigated sperm motility in 301 semen samples of subjects between 30 and 40 years old attempting intra‐cytoplasmic sperm injection/*in vitro* fertilization (ICSI/IVF) (Table 1). Mitochondrial ROS and high membrane potential (MMP), as well as the diaphorase‐type activity of mitochondrial complex I (MCI) in the sperm were examined. Lipid peroxidation and PHB expression in the sperm were also determined. Correlations between mROS, high MMP, lipid peroxidation, PHB expression and sperm motility were then analysed.

**Table 1:** Analysis of the semen parameters of the subjects included in this study (mean ± S.E.M.)

Subject parameters	N (*n* = 114)	A (*n* = 93)	OA (*n* = 94)
Sperm count (×10^6^)	164.8 ± 6.9	109.7 ± 7.1*	14.8 ± 1.2* ^,^ †
Sperm concentration (×10^6^ sperms/ml)	54.9 ± 1.8	33.9 ± 1.6*	5.1 ± 0.3* ^,^ †
Total motility (%)	70.4 ± 0.9	21.8 ± 1.0*	7.1 ± 0.7* ^,^ †
Progressive motility (%)	62.7 ± 0.9	17.4 ± 0.8*	5.4 ± 0.7* ^,^ †
Age (years)	32.1 ± 0.5	32.1 ± 0.6	31.5 ± 0.6

*P < 0.001, compared with N. ^†^ *P* < 0.001, compared with A. N: normospermic; A: asthenospermic; OA: oligoasthenospermic subjects.

## Materials and methods

### Chemicals

All chemicals were purchased from Sigma Chemical Co. (St Louis, MO, USA) unless otherwise stated. PBS solution was purchased from Ambion Inc. (Austin, TX, USA). All fluorescent probes were purchased from Molecular Probes Inc. (Eugene, OR, USA). Rabbit polyclonal antibody to human Prohibitin was from Abcam Inc. (Cambridge, MA, USA) while mouse monoclonal antibody to human β actin from Sigma Chemical Co. Horseradish peroxidase‐conjugated secondary antibody goat anti‐rabbit/mouse IgG was from Santa Cruz Biotechnology, Inc. (Santa Cruz, CA, USA). 3‐[(3‐Cholamidopropyl) dimethylammonio]‐1‐propane sulfonate (CHAPS), urea, thiourea, dithiothuretiol (DTT), and acrylamide were purchased from Amersham Biosciences (Uppsala, Sweden). All buffers were prepared with Milli‐Q water (Millipore, Bedford, MA, USA).

### Sample collection and preparation

The project was approved by the Ethics Committee of Fudan University for Investigations in Humans. Semen samples were collected according to WHO criteria 14 from 301 male subjects between 30 and 40 years old attempting ICSI or IVF at Shanghai JIAI Genetics and IVF Institute of Fudan University after informed consent. The collected samples after liquefaction at 37°C for 30 min. were then subjected to sperm concentration and motility analyses by computer‐assisted sperm analysis assay according to WHO criteria 14. Semen samples collected were identified as normospermic (N, *n* = 114), asthenospermic (A, *n* = 93) or oligoasthenospermic (OA, *n* = 94) subjects, as shown in Table 1. Sperm counts, sperm concentrations and sperm motility were all significantly lower in asthenospermic and oligoasthenospermic subjects (*P* < 0.001; Table 1). Experiments including flow cytometry, enzyme assay and Western blotting were performed in rough semen samples after washing twice with PBS by centrifugation at 300 × g for 10 min. at room temperature (RT). All samples were then cleared of contaminating leucocytes using magnetic Dynabeads^®^ coated with a monoclonal antibody directed against the common leucocyte antigen CD45 (Life Technologies) and confirmed using a zymosan provocation assay 14.

### MitoSOX red assay

The mitochondrial generation of superoxide (mROS) was determined using MitoSOX Red (MSR) that penetrates live cells and selectively localizes to the mitochondria in human sperm 8. Once in this location, the probe is oxidized by superoxide anion, generating a red fluorescence 8. For the assay, the sperm suspensions (10 × 10^6^ cells/ml) were incubated for 15 min. at 37°C with MSR and the vitality stain, SYTOX green, at final concentrations of 2 μM and 0.002 μM respectively. The blank control was performed by incubation without MSR and SYTOX green probes. Sperm samples were then centrifuged at 600 × g for 5 min., and the pellets were re‐suspended in 1 ml PBS and subsequently transferred to 5 ml FACS tubes for flow cytometry analysis. The MSR (red) and SYTOX green (green) fluorescence was then measured on a FACSCalibur flow cytometer (Becton‐Dickinson, San Diego, CA, USA) by argon laser excitation at 488 nm coupled with emission measurements using 530/30 band pass (green) and 585/42 band pass (red) filters. Non‐sperm‐specific events were gated out and 10,000 cells were examined per sample. Data were analysed using CellQuest^™^ software (BD Biosciences). The percentage of cells positive for MSR and SYTOX green probes was determined based upon blank control staining.

### JC‐1 assay

For further detection of high MMP, JC‐1 were applied to report the state of MMP by green for low MMP while red for high MMP. Briefly, the sperm suspensions (1.0 × 10^6^ sperms/ml) were incubated with JC‐1 for 15 min. at a final concentration of 0.25 μM. For the negative control, cells were incubated for 15 min. with 500 μM CCCP (carbonyl cyanide 3‐chlorophenylhydrazone) before JC‐1 staining. The sperm samples were then centrifuged at 600 × g for 5 min., and the sperm pellet was re‐suspended in 1 ml PBS and transferred to a 5 ml FACS tube containing 5 μg/ml propidium iodide (PI) for flow cytometry analysis. The JC‐1 and PI fluorescence was then measured on a FACSCalibur flow cytometer (Becton‐Dickinson) set at 488 nm of argon laser excitation coupled with emission measurements using a N670 long pass filter (far red; PI) to exclude dead cells from the analysis, followed by 530/30 band pass (green; low MMP) and 585/42 band pass (red; high MMP) filters. Non‐sperm specific events were gated out and 10,000 cells were examined per independent sample. The percentage of cells positive for red JC‐1 probe, but negative for PI probe was determined based upon blank control staining.

### Lipid peroxidation assay

Lipid peroxidation was assessed using BODIPY (581/591) C11 as the probe while PI as the viability indicator. This BODIPY C11 probe incorporates into membranes, where it undergoes a shift of the fluorescence emission from 590 nm to 510 nm upon peroxidation by lipid radicals 15. Briefly, the sperm suspensions (1.0 × 10^6^ sperms/ml) were incubated with 2 μM BODIPY C11 for 30 min. at 37°C. The blank control was incubated without BODIPY C11 probe. The samples were then centrifuged at 600 × g for 5 min., and the sperm pellets were re‐suspended in 1 ml PBS. The viability indicator PI (5 μg/ml) was added just before the cells were analysed on a FACSCalibur flow cytometer using an excitation wavelength of 488 nm. The FL‐1 (530/30 nm band pass filter) was used to measure green fluorescence, and FL‐3 (620 nm long pass) was used to measure the shift in red fluorescence upon staining with PI. 10,000 sperm specific events were collected per sample. The percentage of cells positive for green BODIPY C11 and with a shift from red fluorescence upon staining with PI probes was determined after correction for blank control staining.

### Mitochondrial complex I enzyme activity assay

Mitochondrial ROS anions have been shown to be generated from MCI while hydrogen peroxide from mitochondrial complex III in human sperm 8. Based on the above findings, in this study, the diaphorase‐type activity of sperm MCI, independent of ubiquinone, was detected in sperm homogenates using the Complex I Enzyme Activity Microplate Assay Kit (#ab190721; Abcam). The sperm homogenates containing mitochondrial protein was first obtained by a freeze‐thaw technique for mitochondria in hypotonic media 16. Briefly, the sperms were re‐suspended in the hypotonic media containing 20 mM potassium phosphate (pH 7.0) and 5 mM MgCl_2_ at a final concentration of 2 × 10^8^ sperms/ml. The samples were then homogenized using a Dounce (#357538; Wheaton, Millville, NJ, USA) at 4°C followed by the freeze‐thawing the homogenates three times before the enzyme analysis. The total protein concentrations of the supernatant of the homogenates were quantified using the Bradford method (Pierce Biotechnology, Rockford, IL, USA) after centrifugation at 600 × g for 10 min. The enzyme of the MCI is first immunocaptured from the supernatant to the wells of the microplate. The enzyme activity is then determined by the oxidation of NADH to NAD^+^ and the simultaneous reduction in a dye which leads to increased absorbance at 450 nm. The activity is expressed as the change in absorbance (ΔmOD/min) per 100 μg protein of sperm homogenates loaded into the well.

### SDS‐PAGE & Western blotting

The expression level of Prohibitin protein in human sperm from N, A, and OA subjects was measured by Western blot. Briefly, after washing twice with PBS, 10 × 10^6^ sperms were lysed in 200 μl cold lysis buffer (8 M Urea, 2 M Thiourea, 4% CHAPS, 65 mM DTT, 1× proteinase inhibitor cocktail, 1× phosphatase inhibitor cocktail) by sonication for 10 times (each time 3 sec. with a 5‐sec. break) at 35% power on ice and then homogenized on ice for 15 min. before centrifugation (16,000 × g, 15 min., 4°C). The total protein concentrations of the lysates were then quantified using the Bradford method (Pierce Biotechnology). After mixed with 5× protein loading buffer and boiled for 5 min. at 100°C, the cell lysates with 50 μg total proteins extracted from sperm samples were analysed by 12% SDS‐PAGE (one‐dimensional electrophoresis; Bio‐Rad Life Science, Hercules, CA, USA), transferred to PVDF membrane (Millipore) and blocked with 5% nonfat milk in TBST buffer (20 mM Tris, pH 8.0, 150 mM NaCl and 0.1% Tween‐20) for 2 hrs at RT. The membranes were incubated with the primary antibody for Prohibitin (#ab28172; Abcam) at 1:1000 dilution overnight at 4°C, then with a horseradish peroxidase‐conjugated secondary antibody at 1:5000 dilution for 1 hrs at RT, and detected by an enhanced chemiluminescence kit (Amersham Biosciences) according to the manufacturer's instructions. Images were captured using LAS‐3000 (FuJi Film, Tokyo, Japan). Protein molecular weight was estimated by Prestained Protein Ladder (Invitrogen, Carlsbad, CA, USA) with β‐actin as a loading control.

### Statistical analysis

All results are presented as mean ± S.E.M. All variables were checked for normal distribution before statistical analyses (Prism software version 3.0; GraphPad, San Diego, CA, USA). All results were analysed by one‐way anova followed by Newman–Keuls post‐test. In addition, the relationships among percentages of positive sperm with staining of MSR or JC‐1 or BODIPY C11 probes, relative density ratios of Prohibitin expression and sperm motility were analysed by Pearson correlation and linear or nonlinear regression. Probabilities of *P* < 0.05 were considered significant. Each experiment was repeated at least five times.

## Results

### Mitochondrial dysfunction is significantly correlated with human sperm motility loss in asthenospermic or oligoasthenospermic subjects

**Figure 1:**
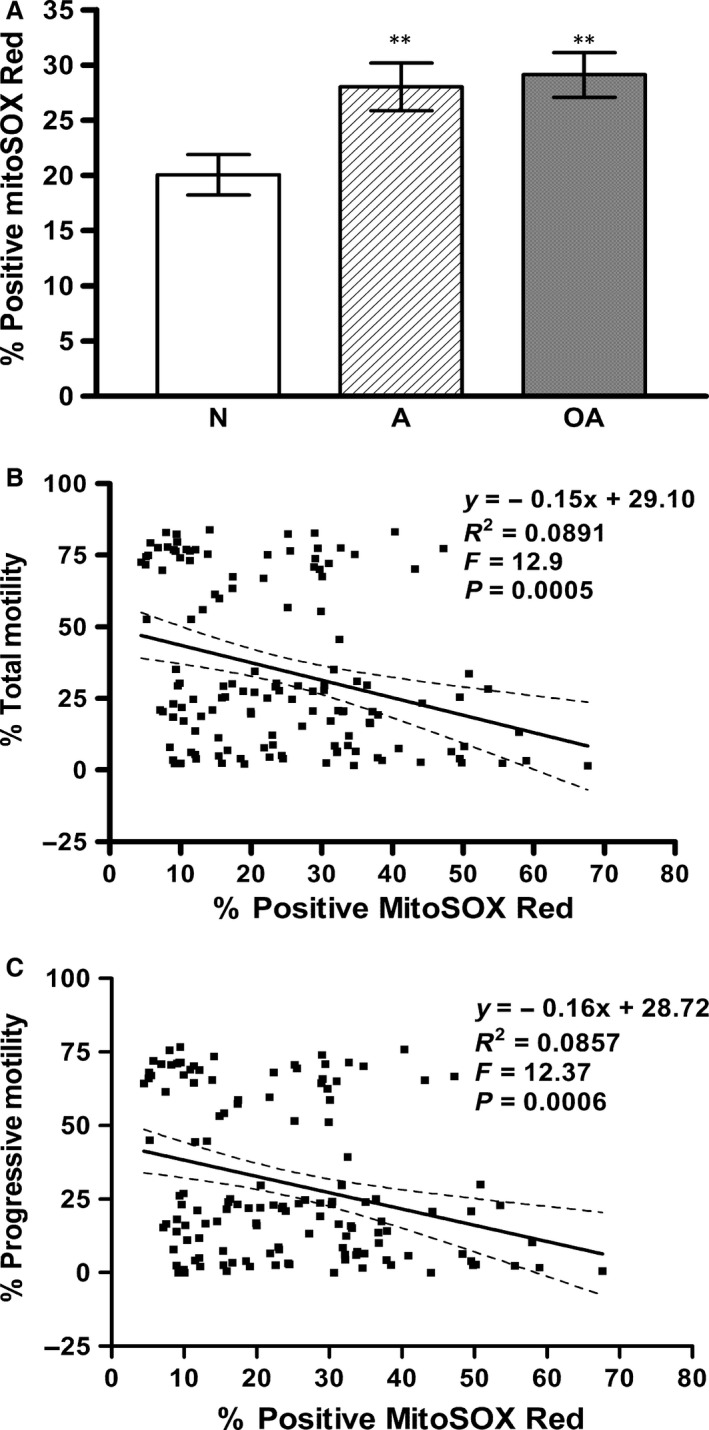
Asthenospermic and oligoasthenospermic men have increased numbers of sperm positive for mitochondrial ROS that modulates sperm motility loss. Sperm mitochondrial ROS (MitoSOX Red), as determined using MitoSOX Red and SYTOX green probes, was analysed using CellQuest^™^ software. The percentage of sperm with both MitoSOX Red and SYTOX green staining was determined by flow cytometric analysis in samples from normospermic (N), asthenospermic (A), and oligoasthenospermic (OA) subjects. (**A**) Histogram demonstrates significant increase, **P < 0.01 compared with N. (**B** and **C**) It shows significantly negative correlations of sperm mitochondrial ROS (MitoSOX Red) with (**B**) the total (*P* < 0.001) and (**C**) progressive (*P* < 0.001) motility of sperm from normospermic (N), asthenospermic (A) and oligoasthenospermic (OA) subjects.

As shown in Figure 1A, sperm mitochondrial ROS, as indicated by the percentage of positive cells with red fluorescence (MSR) in live cells indicated by positive SYTOX green, was significantly higher in sperm from A (28.01 ± 2.16; *P* < 0.01) and OA (29.11 ± 2.03; *P* < 0.01) subjects than in those from N (20.05 ± 1.84) subjects. Correlation analysis showed that mitochondrial ROS was negatively correlated with the total sperm motility (Pearson *r* = −0.2984, *P* = 0.0005; see Fig. 1B) and progressive motility (Pearson *r* = −0.2928, *P* = 0.0006; see Fig. 1C). Moreover, there was a significant linear relationship between mitochondrial ROS and both the total and progressive motility of the sperm (see Fig. 1B and C; *P* < 0.001).

**Figure 2:**
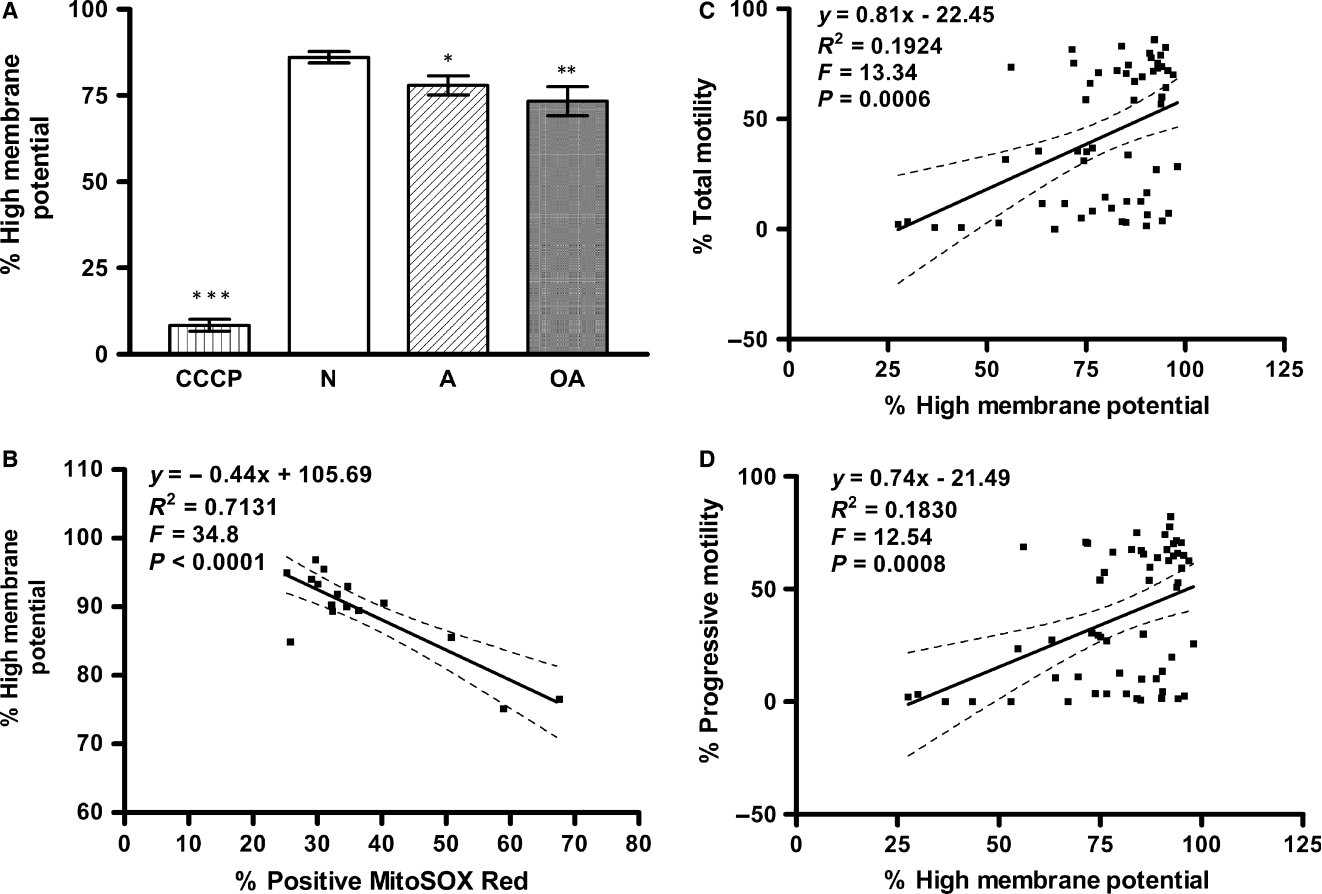
Asthenospermic and oligoasthenospermic men have decreased numbers of sperm positive for high mitochondrial membrane potential that modulates sperm motility loss by increased mitochondrial ROS. The high mitochondrial membrane potential (JC‐1) of sperm, as determined using JC‐1 and PI probes, was analysed using CellQuest^™^ software. The percentage of sperm with JC‐1 staining was determined by flow cytometric analysis in human sperm from normospermic (N), asthenospermic (A), and oligoasthenospermic (OA) subjects. (**A**) Histogram shows a significant decrease, *P < 0.05 compared with N, **P < 0.01 compared with N, ***P < 0.001 compared with N. CCCP was used as a negative control. (**B**–**D**) It shows significantly negative correlation between high membrane potential of sperm and (**B**) sperm mitochondrial ROS (MitoSOX Red) (*P* < 0.0001) whereas positive correlations with (**C**) total motility (*P* < 0.001) and (**D**) progressive motility (*P* < 0.001) of sperm from normospermic (N), asthenospermic (A) and oligoasthenospermic (OA) subjects.

On the other hand, the high membrane potential, as indicated by the percentage of cells positive for the red JC‐1 probe in live cells (negative for the PI probe), was significantly lower in sperm from A (77.94 ± 2.80; *P* < 0.05) and OA (73.35 ± 4.21; *P* < 0.01) subjects than in those from N (86.05 ± 1.62) subjects (Fig. 2A). Correlation analysis showed that mitochondrial high membrane potential was positively correlated with the total sperm motility (Pearson *r* = 0.4386, *P* = 0.0006; see Fig. 2C) and progressive motility (Pearson *r* = 0.4278, *P* = 0.0008; see Fig. 2D), but negatively correlated with mitochondrial ROS (Pearson *r* = −0.8445, *P* < 0.0001; see Fig. 2B). Moreover, there was a significant linear relationship between mitochondrial high membrane potential and both the total and progressive motility of the sperm (see Fig. 2C and D; *P* < 0.001) and mitochondrial ROS (see Fig. 2B; *P* < 0.0001).

### Higher levels of lipid peroxidation are significantly correlated with human sperm motility loss in asthenospermic or oligoasthenospermic subjects

**Figure 3:**
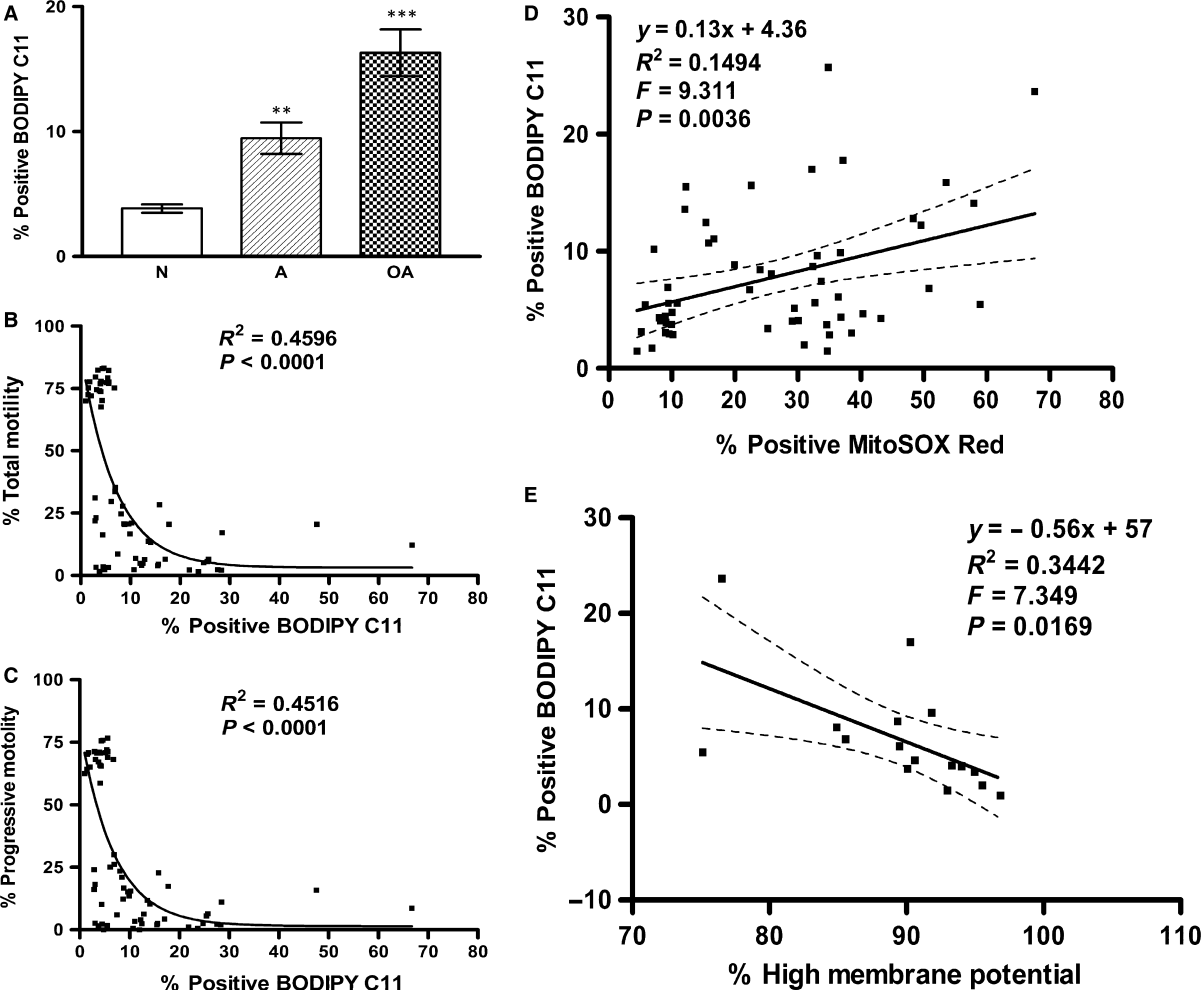
Asthenospermic and oligoasthenospermic men have increased numbers of sperm positive for lipid peroxidation which may modulate sperm motility loss *via* increased mitochondrial ROS and decreased high mitochondrial membrane potential. Lipid peroxidation (BODIPY C11) of sperm, as determined using BODIPY (581/591) C11 and PI probes, was analysed using CellQuest^™^ software. The percentage of sperm with green BODIPY (581/591) C11 staining was determined by flow cytometric analysis in human sperm from normospermic (N), asthenospermic (A), and oligoasthenospermic (OA) subjects. (**A**) Histogram shows significant increase in A (**P < 0.01) compared with N, and OA (***P < 0.001) compared with N or A. (**B**,** C** and **E**) Reveal significantly negative correlations of sperm lipid peroxidation with (**B**) total motility (*P* < 0.0001), (**C**) progressive motility (*P* < 0.0001), and (**E**) high membrane potential of sperm (*P* = 0.0169); whereas there was a positive correlation with (**D**) sperm mitochondrial ROS (MitoSOX Red) (*P* = 0.0036) in normospermic (N), asthenospermic (A) and oligoasthenospermic (OA) subjects.

As shown in Figure 3A, sperm lipid peroxidation levels were significantly higher in sperm from A (9.47 ± 1.26; *P* < 0.01) and OA (16.3 ± 1.88; *P* < 0.001) subjects than in those from N (3.83 ± 0.33) subjects. The increase in the lipid peroxidation levels was greater for the OA subjects than for the A subjects (*P* < 0.001).

Moreover, lipid peroxidation was significantly negatively correlated with mitochondrial high MMP (Pearson *r* = −0.5867, *P* = 0.0169; see Fig. 3E) and total motility (Pearson *r* = −0.4795, *P* < 0.0001; see Fig. 3B) or progressive motility (Pearson *r* = −0.4751, *P* < 0.0001; see Fig. 3C) of the sperm whereas positively with mitochondrial ROS level (Pearson *r* = 0.3866, *P* = 0.0036; see Fig. 3D). Meanwhile, there was a significant nonlinear relationship of lipid peroxidation with both the total and progressive motility of the sperm (see Fig. 3B and C; *P* < 0.0001) or linear relationship of lipid peroxidation with mitochondrial ROS (see Fig. 3D; *P* = 0.0036) and mitochondrial high MMP (see Fig. 3E; *P* = 0.0169).

### No significant difference was detected in the diaphorase‐type activity of MCI in sperm of the groups studied

**Figure 4:**
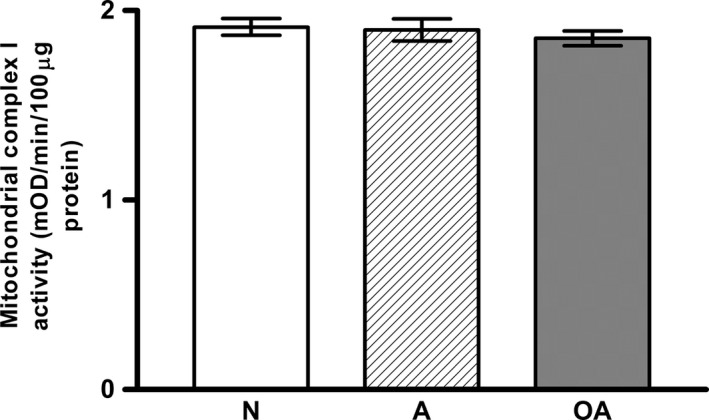
The diaphorase‐type activity of mitochondrial complex I showed no significant change in sperm from asthenospermic and oligoasthenospermic men. The diaphorase‐type activity of mitochondrial complex I was determined by the oxidation of NADH to NAD ^+^ and the simultaneous reduction in a dye which leads to increased absorbance at 450 nm was then analysed. The diaphorase‐type activity of mitochondrial complex I was expressed as the change in absorbance (ΔmOD/min) per 100 μg protein of sperm homogenates loaded into the well. Histogram shows no significant change, *P* > 0.05, compared to N.

As shown in Figure 4, there is no significant difference in the diaphorase‐type activity of MCI in sperm from A (1.898 ± 0.06; *P* > 0.05) or OA (1.854 ± 0.04; *P* > 0.05) subjects compared to that from normospermic (1.913 ± 0.04) subjects.

### PHB expression is significantly negatively correlated with mitochondrial ROS level

**Figure 5:**
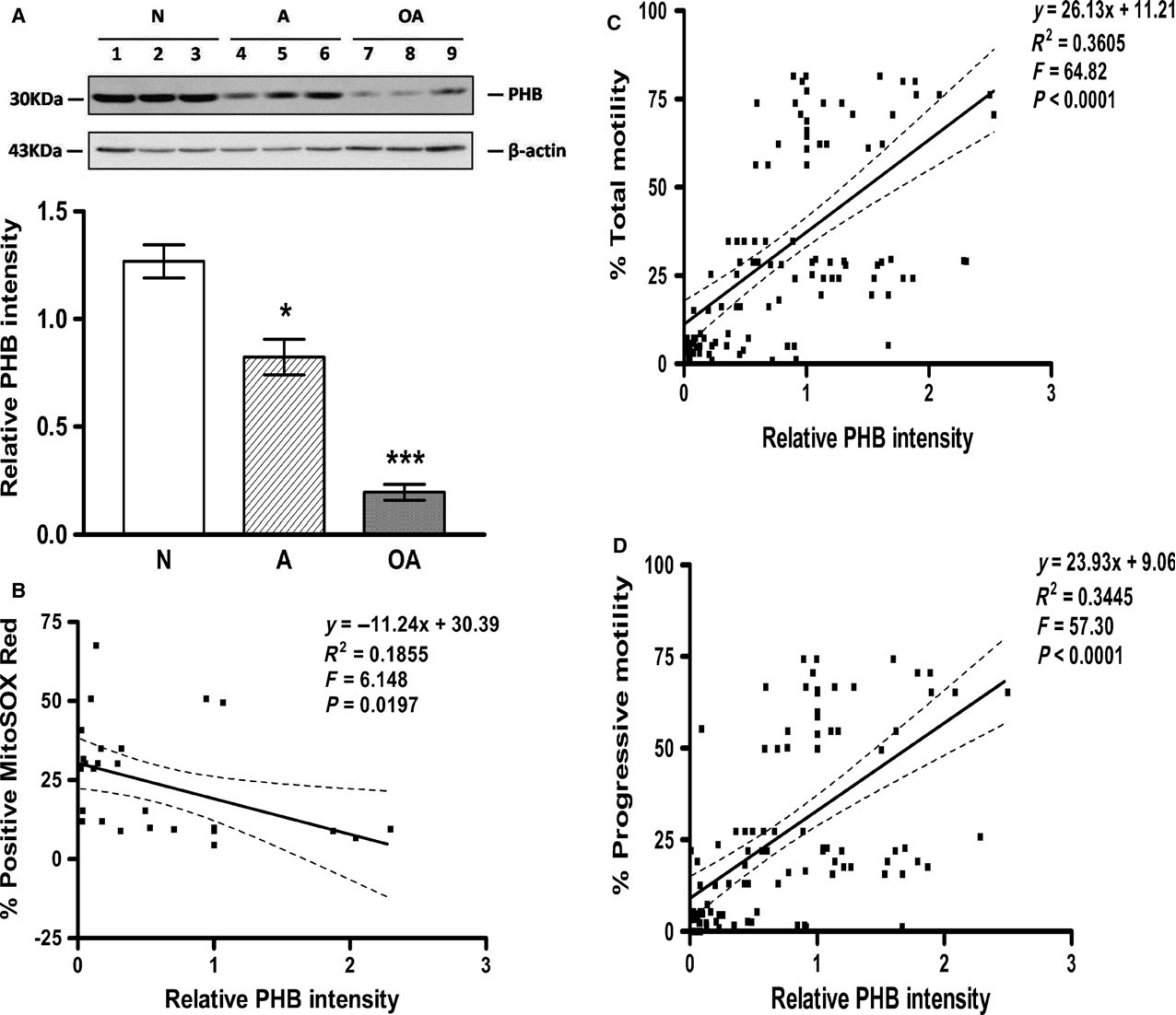
Prohibitin expression is decreased in asthenospermic and oligoasthenospermic men and this decrease correlates with sperm motility and mitochondrial ROS. The expression of PHB protein (30 kD) was analysed by Western blotting in human sperm from normospermic (N), asthenospermic (A) and oligoasthenospermic (OA) subjects using β‐actin as the loading control and densitometric analysis for the relative ratio of PHB protein *versus* β‐actin. Fifty micrograms proteins were loaded in each lane. (**A**) Histogram shows significant differences in Prohibitin, *P < 0.05 compared with N, ***P < 0.001 compared with N or A. (**B**) reveals a significant negative correlation of PHB expression with sperm mitochondrial ROS (MitoSOX Red) (*P* = 0.0197), while there were positive correlations (**C** and **D**) with motility (TM, PR;* P* < 0.0001) in human sperm from normospermic (N), asthenospermic (A) and oligoasthenospermic (OA) subjects.

As shown in Figure 5A, the level of Prohibitin expression was significantly lower in sperm from the A (0.6‐fold of N) and OA (0.15‐fold of N) subjects in comparison to the sperm from the N subjects (see Fig. 5A; *P* < 0.05). Correlation analysis indicated that Prohibitin expression was significantly positively correlated with total motility (Pearson *r* = 0.6004, *P* < 0.0001; see Fig. 5C) and progressive motility (Pearson *r* = 0.5870, *P* < 0.0001; see Fig. 5D) of the sperm. However, it was significantly negatively correlated with mitochondrial ROS levels (Pearson *r* = −0.4307, *P* = 0.0197; see Fig. 5B) in normospermic, asthenospermic and oligoasthenospermic subjects. Meanwhile, there was a significant linear relationship of prohibitin expression with total motility (see Fig. 5C; *P* < 0.0001) and progressive motility (see Fig. 5D; *P* < 0.0001) and mitochondrial ROS (see Fig. 5B; *P* = 0.0197).

## Discussion

Mitochondria of human mature sperm play a key role in the maintenance of sperm motility by the generation of optimal levels of ROS and ATP 8, 14, 15. Mitochondrial membrane potential, created *via* a proton gradient generation during coupling electron transfer at mitochondrial complex I, III and IV, is the key point to promote the conformational change in complex V, resulting in the generation of ATP 17. In this study, sperm of normospermic (N), asthenospermic (A) and oligoasthenospermic (OA) subjects were shown to differ significantly with respect to their total motility (TM), progressive motility (PR), as well as sperm concentration (Table 1). To confirm a decreased MMP level as previously reported 13, JC‐1 was used to further examine the levels of MMP state (high or low). Significantly higher mROS levels (Fig. 1A) and lower high MMP (Fig. 2A) were found in the sperm from the A and OA subjects when compared with those from normospermic (N) subjects. The finding of increased mROS in sperm from infertile men is consistent with the report of Koppers *et al*. 8 in sperm from fertile men after treatment with rotenone (an inhibitor of electron transfer in MCI). Furthermore, a significantly positive correlation between high MMP and the sperm motility (Fig. 2C and D) confirms our recent report 13 and agrees with the finding of Marchetti *et al*. 18 of a positive correlation between high MMP and standard semen parameters such as motility. Meanwhile, significantly negative associations of mROS level with high MMP level (Fig. 2B) and sperm motility (Fig. 1B and C) suggest that high levels of mROS result in lower high MMP, as reported by Treulen 19.

Lipid peroxidation levels are also significantly higher in sperm from A and OA subjects compared with that from normospermic (N) subjects (Fig. 3A), and significantly negatively correlated with sperm motility (Fig. 3B and C). Moreover, a significantly positive correlation is found between mROS and lipid peroxidation levels (Fig. 3D) while there is a negative correlation between high MMP and lipid peroxidation levels (Fig. 3E). These results agree with the recent report of Koppers *et al*. 8, who showed that mROS is generated by defective human spermatozoa and is negatively correlated with motility. Moreover, the induction of ROS on the matrix side of the inner mitochondrial membrane in complex I (MCI) by disruption of mitochondrial electron transport flow resulted in peroxidative damage and a loss of movement in human spermatozoa 8. Taken together, in this study, we further demonstrate that sperm from infertile men are capable of increased production of mROS (superoxide), resulting in lower high MMP, higher percentages of cells with lipid peroxidation and a loss of movement (Figs 1, 2, 3).

Mitochondrial complex I consists of a hydrophobic membrane arm with the hydrophilic peripheral matrix arm. The peripheral matrix arm is composed of two functional modules, an electron input module where MCI binds and oxidizes NADH and generates electrons, and an electron output module where electrons are transported to ubiquinone, the first electron acceptor 20. Coupling electron transfer, protons are translocated across the inner membrane, creating the generation of a proton gradient (a membrane potential) *via* the activity of proton pumping at the membrane arm of MCI. The finding of similar diaphorase‐type activities of MCI in the three groups studied (Fig. 4) directly confirms that the enzyme activity of first functional module of the matrix arm 21, an electron input module where MCI binds and oxidizes NADH and generates electrons, is not affected in sperm from the three groups studied. This finding suggests that a deficiency in electron transfer activity of MCI in the infertile subjects may account for the increase in mitochondrial ROS (superoxide) production, as shown by Ruiz‐Pesini *et al*. 22 who reported a decrease in NADH dehydrogenase activity of MCI at electron transport, measured by monitoring the reduction in ferricyanide in sperm from A subjects.

A possible link between the structural integrity and the functionality of sperm mitochondrial MCI in infertile men may be the mitochondrial PHB complex, that is localized to the inner mitochondrial membrane as a chaperone‐like protein for the maintenance of mitochondrial biogenesis and metabolism including ETC proteins 23. Knockdown of PHB in somatic cells has been shown to result in a block in electron transport at complex I, increased generation of ROS and depolarization of the mitochondrial membrane 12. In this study, the expression of sperm PHB is significantly negatively correlated with the mitochondrial ROS (*P* < 0.05; see Fig. 5B). The results agree with a recent report of Li *et al*. 24 who showed significant down‐regulation of prohibitin under oxidative stress in the rat testis induced by doxorubicin injection. The present report is the first to show the involvement of PHB in mitochondrial ROS generation of human sperm, consistent with the findings in endothelial 12 and Hela cells 25. The mechanism for the increased generation of mitochondrial ROS might be related to a disruption of mitochondrial electron transport flow in complex I of mitochondrial inner membrane, as reported by Koppers *et al*. 8. This may result from decreased sperm PHB expression in infertile asthenospermic and oligoasthenospermic subjects, as seen in endothelial cells after knockdown of PHB 12, and this ultimately results in increased mitochondrial ROS and decreased MMP and sperm motility loss.

**Figure 6:**
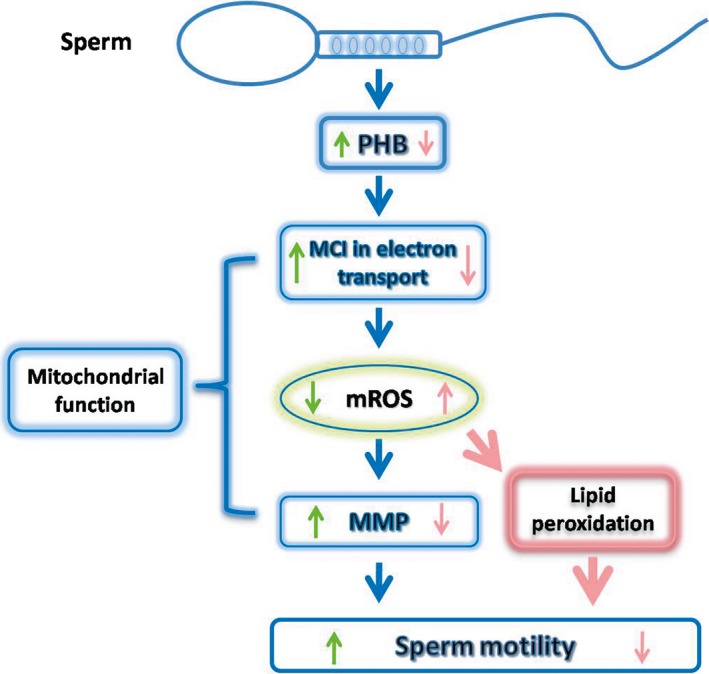
Diagram of the Pathway by which Prohibitin regulates human sperm motility. Under physiological conditions (green arrow), Prohibitin (PHB) maintains sperm motility by regulating mitochondrial functionality, including optimal mROS levels, produced by MCI activity and high MMP. Once PHB is down‐regulated (pink arrow), sperm motility is reduced by dysfunction of mitochondria, such as high mROS by a block of electron transport activity of MCI, low MMP, as well as lipid peroxidation. mROS, mitochondrial ROS; MCI, mitochondrial complex I; MMP, mitochondrial membrane potential.

In conclusion, this study demonstrates that mitochondrial ROS levels are significantly higher in sperm from A and OA subjects than those from normospermic (N) subjects, and this may be related to a block in electron transport at complex I. This occurs in the presence of decreased PHB expression, ultimately resulting in depolarization of the mitochondrial membrane, lipid peroxidation and a decrease in sperm motility (Fig. 6).

## Conflict of interest

The authors confirm that there are no conflicts of interest.
